# Indomethacin Abolishes the Potentiation Effect of Testosterone on the Relaxation Induced by Salbutamol and Theophylline by Directly Blocking the K^+^ Channels in Airway Smooth Muscle

**DOI:** 10.3390/molecules30112259

**Published:** 2025-05-22

**Authors:** Jorge Reyes-García, Abril Carbajal-García, Verónica Díaz-Hernández, María F. Casas-Hernández, Luis M. Montaño

**Affiliations:** 1 Departamento de Farmacología, Facultad de Medicina, Universidad Nacional Autónoma de México, Mexico City 04510, Mexico; reyes.garcia.jorge@gmail.com (J.R.-G.); carbajalabril@gmail.com (A.C.-G.);; 2 Departamento de Embriología, Facultad de Medicina, Universidad Nacional Autónoma de México, Mexico City 04510, Mexico

**Keywords:** airway smooth muscle, testosterone, salbutamol, theophylline, indomethacin, ibuprofen, acetylsalicylic acid

## Abstract

Indomethacin, ibuprofen, and acetylsalicylic acid (ASA) are non-steroidal anti-inflammatory drugs (NSAIDs) that inhibit prostaglandin (PG) synthesis. Previous studies in airway smooth muscle demonstrated that chronic exposure to testosterone (TES, 40 nM) enhances the relaxation induced by salbutamol and theophylline due to K^+^ channel increment, without modifying cyclooxygenase expression. This study examines how indomethacin, ibuprofen, and ASA affect K^+^ currents and the relaxation response to these bronchodilators. In organ baths, tracheas from young male guinea pigs chronically (48 h) treated with 40 nM TES showed increased relaxation to salbutamol and theophylline, which was completely abolished by indomethacin. Patch-clamp recordings revealed that TES increased salbutamol- and theophylline-induced K^+^ currents, and only indomethacin fully inhibited this potentiation; ibuprofen and ASA had partial effects. The involved currents included voltage-dependent K^+^ (K_V_) and high-conductance Ca^2+^-activated K^+^ (BK_Ca_) channels. Our results demonstrate that indomethacin exerts a dual action, inhibiting K^+^ channel activity and PG synthesis, unlike ibuprofen and ASA. This dual mechanism explains its stronger inhibitory effect on TES-enhanced ASM relaxation. These findings suggest that indomethacin may counteract the protective effects of TES, which promotes anti-inflammatory and smooth muscle-relaxing states. Therefore, it is advisable to exercise caution when prescribing indomethacin to young males with asthma, as the protective role of TES may diminish, potentially resulting in an exacerbation of asthma symptoms.

## 1. Introduction

Non-steroidal anti-inflammatory drugs (NSAIDs), such as indomethacin, ibuprofen, and acetylsalicylic acid (ASA), commonly known as aspirin, are among the most widely used pharmacological medications around the world due to their anti-inflammatory, antipyretic, and analgesic properties [1,2,3]. Indomethacin is a potent nonselective inhibitor of cyclooxygenase (COX)-1 and COX-2, primarily acting by suppressing the production of prostaglandins (PGs) [4,5,6]. Ibuprofen, also a nonselective COX inhibitor, is generally considered to have a weaker activity compared to indomethacin or other NSAIDs [6]. Aspirin also inhibits both COX-1 and COX-2 enzymes; however, unlike indomethacin and ibuprofen, which bind to cyclooxygenases reversibly, aspirin forms an irreversible bond [3]. Additionally, this drug inhibits thromboxane A_2_ synthesis in platelets, thereby reducing platelet aggregation [7]. As the primary mechanism of these medications involves the inhibition of prostanoid synthesis, their widespread use in managing fever, pain, and inflammation is well justified [2,8].

Prostaglandins are crucial in regulating the airway smooth muscle (ASM) tone [9,10,11,12,13]. For instance, prostaglandin E_2_ (PGE_2_), released by the airway epithelium, is a bronchoprotective metabolite [11,14,15]. The broncho-relaxant effects of this lipid substance have been extensively studied in animal models and humans, using in vitro and in vivo approaches [9,13,16,17,18]. ASM relaxation induced by PGE_2_ is attributed to activating the E-type prostanoid receptors 2 and 4 (EP_2_ and EP_4_). Both receptors are coupled to a G_S_ protein, which leads to the signaling of 3′,5′-cyclic adenosine monophosphate (cAMP) and protein kinase A (PKA) [17,19,20,21]. Indomethacin has been widely used to investigate the involvement of prostaglandins in the regulation of airway tone and reactivity, thereby contributing to a better understanding of their role in respiratory conditions such as asthma [13,22,23,24,25].

Previous studies conducted by our research group indicated that testosterone (TES) enhanced the relaxation of the guinea pig ASM induced by salbutamol and theophylline, both commonly used drugs for relieving bronchospasm in asthmatics. This enhancement was linked to a genomic pathway produced by the androgen that increased expression of the β_2_ adrenergic receptor and voltage-dependent K^+^ channels (K_V_), specifically K_V_1.2 and K_V_1.5 [26,27]. We also found that TES increased ATP-elicited ASM relaxation via increased K_V_ channels [28]. Salbutamol, ATP, and theophylline induce the cAMP-PKA signaling cascade and open K_V_ and high-conductance Ca^2+^-activated K^+^ channels (BK_Ca_). We have also demonstrated that TES caused the relaxation of guinea pig ASM. However, indomethacin reduced this relaxing effect, suggesting that PGs could be involved in this process [24]. In this regard, TES may regulate PGs production in different tissues, including the ASM [24,29,30].

Considering the hypothesis that TES may increase PGE_2_ synthesis in airways, we conducted preliminary experiments showing that indomethacin effectively blocks the androgen-induced effects on salbutamol-elicited relaxation. In this context, we explored the possibility that TES could modulate one of the cyclooxygenase isoforms to increase PGE_2_ production and enhance ASM relaxation. However, recent studies have indicated that TES does not increase the expression of either COX-1 or COX-2 [28]. Therefore, the aim of the present study is to elucidate the mechanism by which indomethacin counteracts the genomic effects of TES on airway relaxation. Specifically, we seek to determine whether indomethacin blocks the TES-mediated potentiation of salbutamol- and theophylline-induced relaxation by interfering with prostaglandin production despite unchanged COX expression. In addition, we investigated whether this inhibitory effect involves a secondary mechanism related to the blockade of K^+^ channels, which are essential components of the signaling cascade triggered by salbutamol and theophylline to promote ASM relaxation. To further clarify the specificity of this mechanism, we also examined whether ibuprofen or aspirin affects the salbutamol-induced K^+^ currents, allowing us to compare their effects with those of indomethacin and better understand the drug’s action.

## 2. Results

### 2.1. Influence of Indomethacin on the Enhanced Relaxation Response to Salbutamol and Theophylline

**Figure 1:**
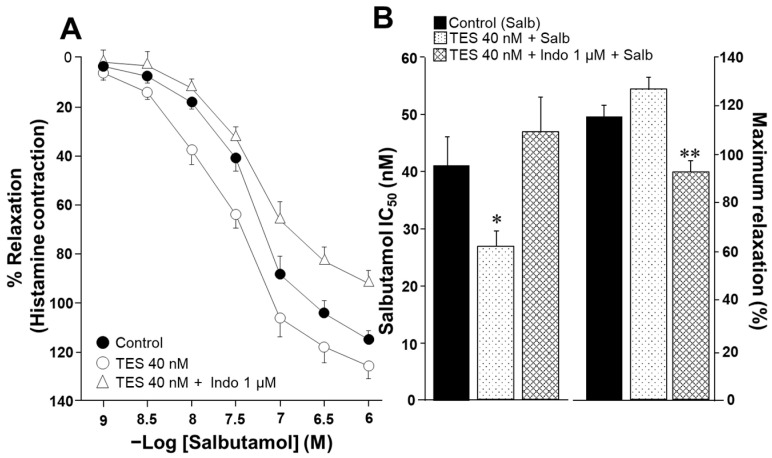
Salbutamol-induced relaxation of guinea pig tracheal rings is enhanced by testosterone (TES) following 48 h of incubation. However, the potentiation effect of TES is blocked by the administration of indomethacin. (**A**) The cumulative concentration curve for salbutamol (1, 3.2, 10, 32, 100, 320, and 1000 nM) effectively relaxes tracheal rings pre-contracted with 10 µM histamine. When the tissues are incubated with 40 nM TES for 48 h, the concentration–response curve for salbutamol shifts to the left, indicating an increased response. The enhancement of the salbutamol response by TES is eliminated when indomethacin (Indo 1 µM, administered 1 h before the salbutamol curve) is given. (**B**) Bar graphs illustrate that, compared to the control group, the 40 nM TES significantly lowers the salbutamol (Salb) inhibitory concentration 50% (IC_50_). In contrast, Indo increases this pharmacodynamic parameter. Furthermore, Indo significantly decreases the maximal response to Salb when the tracheal tissues have been treated with TES, suggesting that this drug may play a role in the androgen response. Bars and symbols stand for mean ± the standard error of the mean (SEM), * *p* < 0.05, ** *p* < 0.01, *n* = 8. Dunnett’s multiple comparison tests were conducted after repeated-measure analyses of variance.

**Figure 2:**
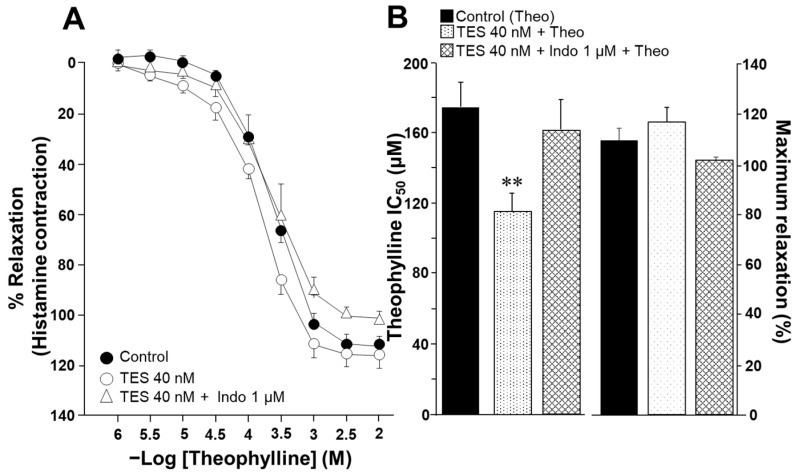
Chronic exposure to testosterone (TES) enhances the relaxation of guinea pig tracheal smooth muscle induced by theophylline, and this effect is eliminated with the treatment of indomethacin. (**A**) Theophylline (at concentrations of 1, 3.2, 10, 32, 100, 320, and 1000 µM, and 3.2 mM and 1 mM) induces relaxation in tracheal rings precontracted with histamine (10 µM). After a 48 h preincubation with TES (40 nM), the concentration–response curve for theophylline shifts to the left. The addition of 1 µM indomethacin (Indo), a nonselective COX inhibitor, removes the effect of TES. (**B**) Compared to the control group, the bar graph shows that TES decreases the inhibitory concentration 50% (IC_50_) of theophylline (Theo), while Indo reverses the effect of the androgen. The maximal response of tracheal tissues to Theo remains consistent across all groups. Symbols and bars represent the mean ± SEM, ** *p* < 0.01, *n* = 7. Dunnett’s multiple comparison tests were conducted after repeated-measure analyses of variance.

To evaluate the effect of indomethacin on the ASM responsiveness to salbutamol and theophylline, isolated tracheal rings from guinea pigs were precontracted with 10 µM histamine until a stable plateau was achieved. Cumulative concentrations of each bronchodilator were then administered. In a subset of samples, tissues were pretreated with testosterone (TES) at a concentration of 40 nM for 48 h. Notably, TES was no longer present when the tissues were mounted in organ bath chambers. Consistent with our previous findings, TES significantly increased the relaxation induced by salbutamol and theophylline [26,27]. However, this effect was reversed upon exposure to 1 µM indomethacin. As shown in Figure 1A, salbutamol induced ASM relaxation in a concentration-dependent manner. TES at 40 nM caused a leftward shift in the concentration–response curve, whereas indomethacin completely abolished this effect. The inhibitory concentration 50% (IC_50_) value for salbutamol, which is the concentration required to achieve half-maximal relaxation, was significantly lower for the TES-treated group compared to the control group (26.69 ± 2.7 nM vs. 41.07 ± 5.16 nM). However, the IC_50_ values from tissues treated with indomethacin one hour before the relaxation curve showed no significant differences compared to the control group (46.72 ± 6.30 nM vs. 41.07 ± 5.16 nM). Nonetheless, we observed a significant reduction in maximal relaxation responses in tissues incubated with indomethacin (Figure 1B). In addition, the IC_50_ values for theophylline were markedly lower in tracheal tissues exposed to the androgen (118.92 ± 9.43 µM vs. 170.03 ± 13.6 µM). In contrast, tracheal rings treated with indomethacin showed no significant difference in IC_50_ values compared to controls (150.89 ± 14.58 µM vs. 170.03 ± 13.6 µM), a pattern similar to that observed with salbutamol (Figure 2). The presence of indomethacin abolished the TES-induced effect. Finally, no significant differences were observed in the maximal relaxation response between the control and TES groups (110.47 ± 4.74% vs. 115.83 ± 5.77%, respectively).

### 2.2. Indomethacin Attenuates the Testosterone-Mediated Enhancement of K^+^ Currents Induced by Salbutamol and Theophylline

**Figure 3:**
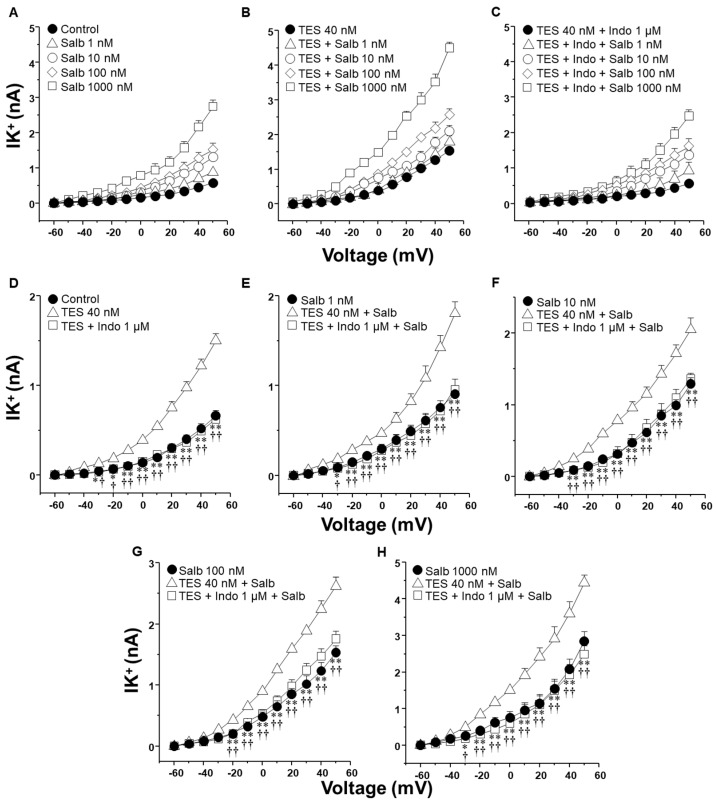
Indomethacin reverts the enhancement of salbutamol-induced K^+^ currents elicited by testosterone (TES) in guinea pig ASM cells. (**A**) To induce outward K^+^ currents (IK^+^), single cells were subjected to a series of depolarization steps from −60 to +50 mV in increments of 10 mV. The IK^+^ augmented in tracheal myocytes perfused with salbutamol (Salb) at increasing concentrations 1–1000 nM (*n* = 8). (**B**) Smooth muscle cells treated with 40 nM TES for 48 h had higher salbutamol-induced IK^+^ than the control group (*n* = 8). (**C**) This effect was eliminated by indomethacin (Indo 1 µM, nonselective COX inhibitor; *n* = 8). (**D**–**H**) The three experimental groups have their individual Salb concentration statistical analysis tested. According to the data, Salb and depolarizing pulses increased IK^+^ in cells exposed to TES 40 nM compared to the control group (without TES). Indo abolished this effect. Various scales were applied to the *Y*-axis in (**D**–**H**) to display the statistical differences. Dunnett’s test was performed after an analysis of variance. Symbols represent the mean values ± SEM. In panel (**D**), * *p* < 0.05, ** *p* < 0.01 comparing control vs. TES group; † *p* < 0.05, †† *p* < 0.01 comparing TES + Indo group vs. TES group. In panels (**E**–**H**), * *p* < 0.05, ** *p* < 0.01 comparing Salb group vs. TES + Salb group; † *p* < 0.05, †† *p* < 0.01 comparing TES + Indo + Salb group vs. TES + Salb group.

**Figure 4:**
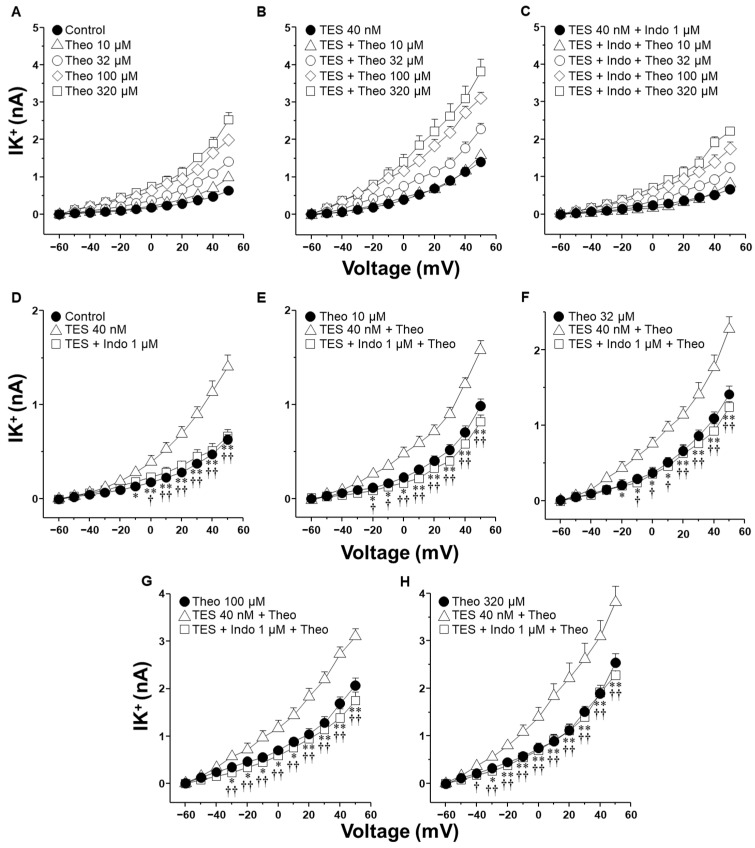
Indomethacin annulled testosterone (TES) augmented K^+^ currents (IK^+^) induced by theophylline (Theo). (**A**) In guinea pig ASM cells, Theo (10–320 µM) elicited a concentration-dependent increase in IK^+^ (*n* = 7). (**B**) Chronic pre-treatment with TES (40 nM for 48 h) augmented Theo-induced IK^+^ (*n* = 6). (**C**) Indomethacin (Indo, 1 µM) preincubation for 10 min before Theo abolished the TES potentiation effect (*n* = 6). For every tested Theo concentration, including experiments with only TES and Indo, statistical analysis is shown in panels (**D**–**H**). Symbols represent mean ± SEM. Analysis of variance was conducted, followed by Dunnett’s multiple comparison tests. In figure (**D**), * *p* < 0.05, ** *p* < 0.01 comparing control vs. TES groups; † *p* < 0.05, †† *p* < 0.01 comparing TES + Indo vs. TES groups. For the remaining figures (**E**–**H**), * *p* < 0.05, ** *p* < 0.01 comparing Theo vs. TES + Theo groups; † *p* < 0.05, †† *p* < 0.01 comparing TES + Indo + Theo vs. TES + Theo groups.

The activation of K^+^ channels plays a crucial role in the ASM relaxation induced by salbutamol and theophylline. Patch-clamp recordings were performed in cultured airway myocytes to investigate the potential modulatory effect of indomethacin on this process. Depolarizing voltage steps evoked voltage-dependent outward K^+^ currents (IK^+^). Perfusion of the cells with increasing concentrations of salbutamol (1–1000 nM) or theophylline (10–320 µM) resulted in a pronounced, concentration-dependent enhancement of the IK^+^ (Figure 3A and Figure 4A). The interval between administering the first and last doses of salbutamol or theophylline was ~40 min, indicating that tracheal myocytes were exposed to one of these bronchodilators for this duration. Myocytes incubated with TES 40 nM showed enhanced IK^+^ in response to salbutamol or theophylline (Figure 3B and Figure 4B). Perfusing indomethacin 1 µM for 10 min before stimulating the myocytes reversed the TES-enhancement of the IK^+^ following stimulation with either bronchodilator (Figure 3C and Figure 4C). To properly assess the modulatory effect of indomethacin on TES-enhanced K^+^ currents, the increment in the IK^+^ was evaluated according to the concentrations of salbutamol or theophylline applied. Figure 3D and Figure 4D demonstrate that TES significantly enhanced the IK^+^ triggered by the depolarizing pulse protocol from steps −30 mV and −10 mV ahead, respectively, while indomethacin abolished this effect. The addition of salbutamol (1–1000 nM) or theophylline (10–320 µM) amplified the IK^+^ during the depolarizing step protocol (Figure 3E–H and Figure 4E–H). Furthermore, TES markedly increased the salbutamol- and theophylline-induced IK^+^ from −30 mV (Figure 3E,F,H and Figure 4G,H) or −20 mV (Figure 3G and Figure 4E,F). Indomethacin counteracted this effect. In summary, these findings corroborate our previous reports that TES enhances the activity of K^+^ channels. Additionally, the data point out that indomethacin may exert an inhibitory effect on these channels. 

### 2.3. Ibuprofen Partially Reduced the Testosterone-Mediated Enhancement of K^+^ Currents Induced by Salbutamol

**Figure 5:**
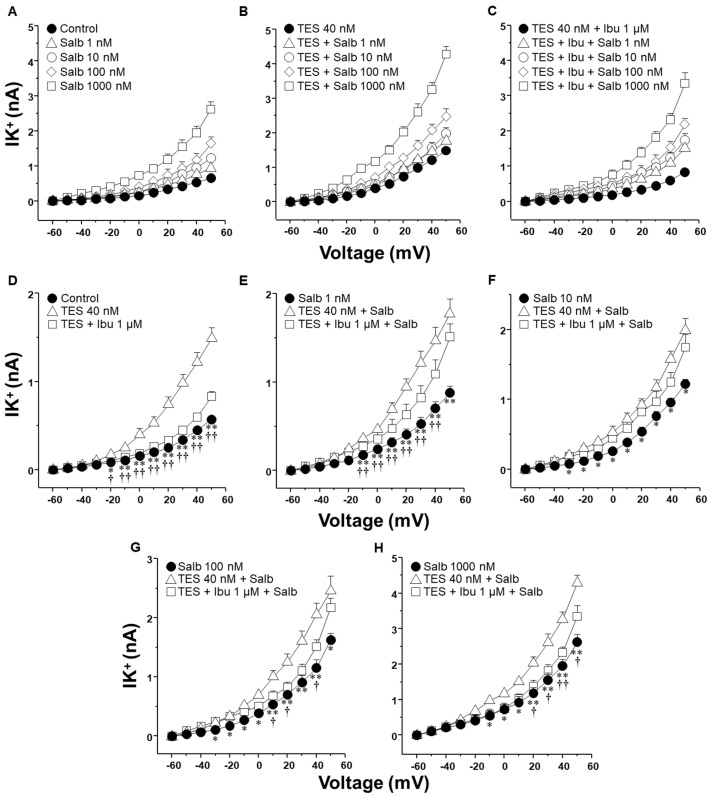
Ibuprofen partially reduced the effect of testosterone (TES) on the increased K^+^ currents (IK^+^) evoked with salbutamol (Salb) in ASM. (**A**) A series of depolarization steps from −60 to +50 mV and applying Salb (1–1000 nM) to single airway myocytes markedly increased the IK^+^ (*n* = 7). (**B**) The chronic incubation of TES (40 nM, 48 h) enhanced the Salb-triggered rise in IK^+^ (*n* = 7). (**C**) The perfusion of 1 µM ibuprofen (Ibu, a nonselective COX inhibitor) for 10 min prior to Salb stimulation diminished the TES-induced enhancement in IK^+^ (*n* = 7). Panels (**D**–**H**) exhibit statistical analysis for each concentration of Salb examined, including experiments conducted with TES alone and Ibu. It is important to note that Ibu abolishes the TES effect on the increase in IK^+^ induced by 1, 100, 1000 nM of Salb. Symbols represent mean ± SEM. In panel (**D**), * *p* < 0.05, ** *p* < 0.01 when comparing control group vs. TES group; † *p* < 0.05, †† *p* < 0.01 comparing TES + Ibu group vs. TES group. In panels (**E**–**H**), * *p* < 0.05, ** *p* < 0.01 when comparing Salb vs. TES + Salb groups; † *p* < 0.05, †† *p* < 0.01 comparing TES + Ibu + Salb group vs. TES + Salb group. A one-way ANOVA was conducted, followed by Dunnett’s tests.

We also investigated the effect of ibuprofen and aspirin, two of the most commonly used NSAIDs, on the IK^+^ triggered by salbutamol. As illustrated in Figure 5, TES markedly enhanced salbutamol-induced IK^+^, while ibuprofen (1 µM) partially attenuated this effect across nearly all concentrations of salbutamol tested. Specifically, Figure 5E illustrates that TES amplified the 1 nM salbutamol-induced IK^+^ starting from −10 mV, whereas ibuprofen nullified this effect between −10 mV to 40 mV. Figure 5F shows that TES significantly increased the IK^+^ from −10 mV onward, but ibuprofen did not modify the influence of TES on the IK^+^ at any of the voltages assessed. In Figure 5G, it is evident that ibuprofen only reduced the TES-enhanced K^+^ current at 10, 20, and 40 mV. Moreover, Figure 5H illustrates that ibuprofen completely blocked the TES-augmented K^+^ current at 20, 30, 40, and 50 mV.

### 2.4. Aspirin Blocks the Testosterone-Mediated Enhancement of K^+^ Currents Induced by Salbutamol but Require Time to Do It

**Figure 6:**
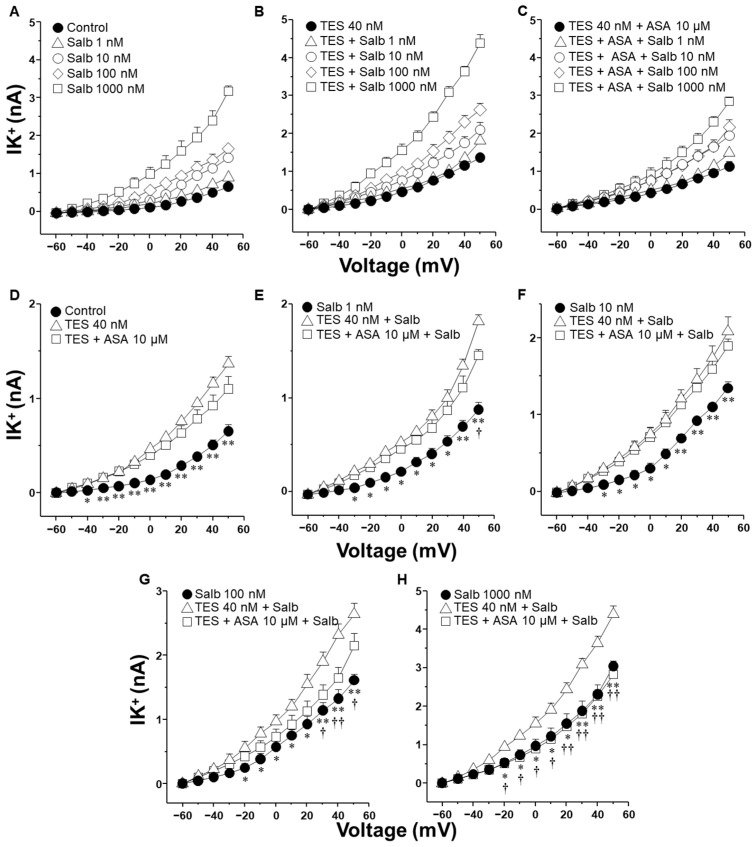
Testosterone (TES) incubated for 48 h in guinea pig tracheal myocytes enhances the salbutamol-induced IK^+^, while acetylsalicylic acid (ASA) reverses this androgenic effect when myocytes are stimulated with 100 or 1000 nM salbutamol. (**A**) IK^+^ was elicited by a series of depolarizing pulses ranging from −60 to +50 mV in 10 mV increments. These pulses evoked IK^+^ currents peaking at approximately 0.5 nA. Stimulation with salbutamol (Salb, 1–1000 nM) increased IK^+^ in a concentration-dependent manner, reaching values around 3 nA. (**B**) TES 40 nM potentiated the IK^+^ induced by depolarizing pulses at all concentrations of salbutamol tested, with currents reaching approximately 4 nA. In (**C**) is illustrated the effect of ASA (10 µM) on the TES-induced enhancement of IK^+^. (**D**–**H**) illustrate the individual effects of the different salbutamol concentrations on the IK^+^, along with their modulation by TES and ASA. (**D**) TES significantly increased IK^+^ from −40 mV onward, while ASA did not modify it. (**E**) Perfusion with salbutamol (1 nM) elicited IK^+^ peaking around 1 nA. TES further increased the IK^+^ from −30 mV, but ASA had no significant effect until 50 mV. (**F**) In cells stimulated with salbutamol 10 nM, TES enhanced IK^+^ from −30 mV. ASA did not alter this androgenic effect. (**G**) Salbutamol 100 nM induced IK^+^ peaking at ~1.5 nA in control conditions; TES potentiated this effect from −20 mV onward. However, ASA reversed the TES-induced potentiation from +30 mV. (**H**) In control cells, salbutamol 1000 nM induced IK^+^ peaking at ~3 nA. TES markedly augmented the current from −20 mV, an effect that was completely abolished by ASA. Different *Y*-axis scales were used to clearly present statistical comparisons. Data are expressed as mean ± SEM. One-way ANOVA followed by Dunnett’s post hoc test was used for statistical analysis. * *p* ≤ 0.05, ** *p* ≤ 0.01 comparing control vs. TES group, or Salb vs. Salb + TES group; † *p* ≤ 0.05, †† *p* ≤ 0.01 when comparing TES + ASA + Salb vs. TES + Salb group, *n* = 6.

Figure 6 summarizes the impact of aspirin on the IK^+^ induced by salbutamol. As shown in the previous figures, TES enhanced the IK^+^ evoked by depolarizing pulses and all concentrations of salbutamol used (Figure 6D–H). However, aspirin 10 µM significantly attenuated the salbutamol-induced IK^+^ at concentrations of 100 nM and 1000 nM, with the inhibitory effect becoming evident at depolarizing potentials of 30 mV and −20 mV, respectively (Figure 6G,H). The time required to initially observe this phenomenon was ~40 min. Taken together, these findings indicate that ibuprofen can partially reduce the TES-enhanced K^+^ currents. However, the inhibitory effect observed with indomethacin is significantly more pronounced. This stronger effect likely reflects a dual mechanism of action: first, indomethacin seems to block the K^+^ channels directly; second, it inhibits the synthesis of PGE_2_, which appears to contribute to the potentiation of K^+^ currents by TES. In contrast, aspirin required time to induce its effects on K^+^ currents.

### 2.5. Indomethacin but Not Ibuprofen and Aspirin Attenuate the Testosterone-Mediated Enhancement of K^+^ Currents When Salbutamol Is Applied in a Unique Concentration

**Figure 7:**
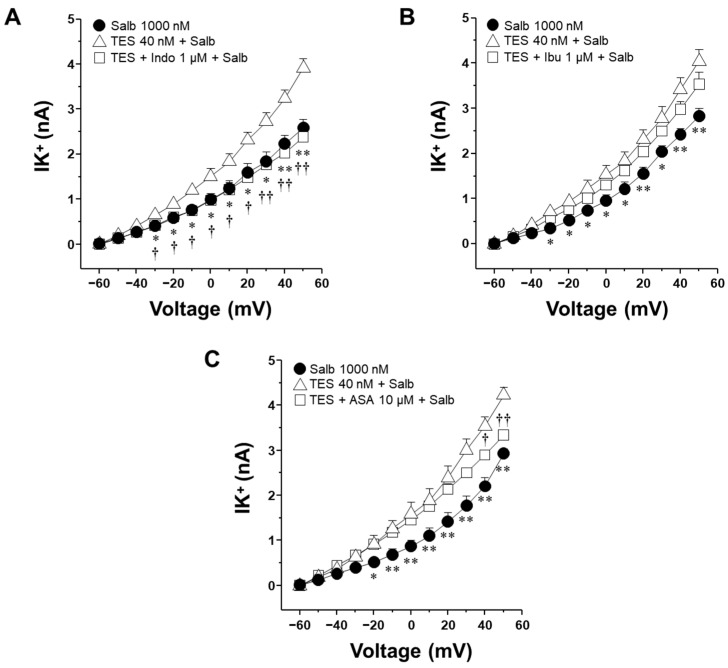
Indomethacin, but not ibuprofen or aspirin, inhibits salbutamol-induced K^+^ currents enhanced by testosterone in airway smooth muscle cells. Airway myocytes were pretreated with testosterone (TES, 40 nM) for 48 h and subsequently stimulated with salbutamol (1000 nM, 5 min) in the presence of nonsteroidal anti-inflammatory drugs (NSAIDs): indomethacin (Indo, 1 µM), ibuprofen (Ibu, 1 µM), or aspirin (ASA, 100 µM). (**A**) Indomethacin attenuated the TES-induced enhancement of salbutamol-evoked outward K^+^ currents (IK^+^) from −30 mV onwards. (**B**) Ibuprofen did not significantly affect these currents under the same conditions. (**C**) Aspirin significantly attenuated the enhancement of K^+^ at 40 and 50 mV. These findings suggest a distinct inhibitory effect of indomethacin on TES-enhanced IK^+^, potentially through direct regulation of the K^+^ channels activity. Data are expressed as mean ± SEM. One-way ANOVA followed by Dunnett’s post hoc test, was used for statistical analysis. * *p* ≤ 0.05, ** *p* ≤ 0.01 comparing Salb vs. TES + Salb group; † *p* ≤ 0.05, †† *p* ≤ 0.01 when comparing TES + Indo/ASA + Salb vs. TES + Salb group.

Chronic exposure (24 h) to salbutamol or cAMP, its downstream effector, promotes the production of PGE_2_ in ASM cells [31]. To investigate whether PGE_2_ contributes to the enhancement of the IK^+^ induced by salbutamol, and its subsequent blockade by NSAIDs, ASM cells pretreated with TES were stimulated with the highest concentration of salbutamol (1000 nM, for 5 min) in the presence of indomethacin, ibuprofen, or ASA. Indomethacin attenuated the enhancement of the salbutamol-elicited IK^+^ that the androgen caused from −30 mV onwards (Figure 7A). In contrast, neither ibuprofen nor aspirin caused a comparable effect on these currents (Figure 7B,C). Aspirin significantly decreased TES-enhanced IK^+^ at 40 and 50 mV, while ibuprofen had no discernible impact. The absence of inhibition by ibuprofen or aspirin indicates that their effect on salbutamol-induced IK^+^ observed in Figure 5 and Figure 6 may primarily result from the suppression of PGE_2_ production.

### 2.6. Indomethacin’s Effect on K^+^ Currents, Enhanced by TES, Primarily Involves Voltage-Dependent K^+^ Channels and, to a Lesser Extent, High-Conductance Ca^2+^-Activated K^+^ Channels

**Figure 8:**
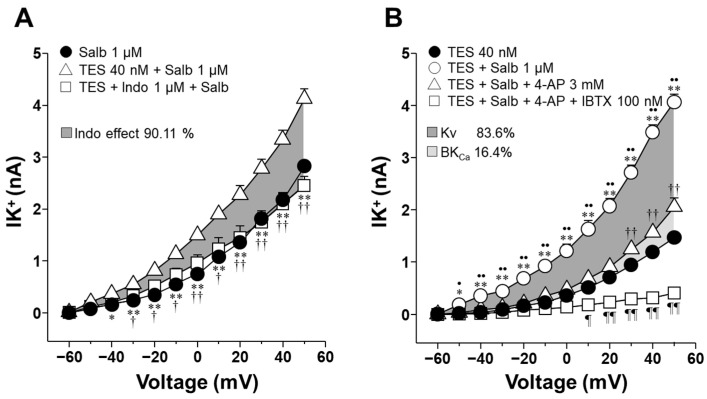
Indomethacin annuls the augmented salbutamol-induced K^+^ currents (IK^+^) by blocking voltage-dependent K^+^ channels (K_V_) and high-conductance Ca^2+^-activated K^+^ channels (BK_Ca_) in airway myocytes treated with testosterone (TES). (**A**) Depolarizing pulses from −60 to +50 mV and 1 µM salbutamol (Salb) increased the IK^+^ in tracheal myocytes treated with 40 nM TES. Indomethacin (Indo, 1 µM), a COX inhibitor, significantly reduced the Salb-induced IK^+^ from −30 mV ahead (*n* = 6). The dark gray area illustrates the area under the curve that the Indo is responsible for 90.11% of modifying the TES-induced enhancement of IK^+^. (**B**) IK^+^ triggered by depolarizing pulses and Salb were higher in myocytes incubated with 40 nM TES (48 h). The androgen effect was nullified by 3 mM 4-aminopyridine (4-AP, K_V_ blocker) and iberiotoxin (IBTX, 100 nM), a selective blocker of the BK_Ca_. The dark gray region of the area under the curve indicates that the K_V_ subtype accounts for 83.6% of the TES-induced enhancement of IK^+^, while BK_Ca_ contributes 16.4%, as shown in the light gray region (*n* = 7). These results point out that indomethacin may directly block both types of K^+^ channels, thereby reducing the TES enhancement of IK^+^. Symbols represent mean ± SEM. Analyses of variance using repeated measures were conducted, followed by Dunnett or Student–Newman–Keuls multiple comparison tests. In figure (**A**), * *p* < 0.05, ** *p* < 0.01 comparing Salb vs. TES + Salb groups; † *p* < 0.05, †† *p* < 0.01 comparing TES + Indo + Salb vs. TES + Salb groups. In figure (**B**), * *p* < 0.05, ** *p* < 0.01 comparing TES + Salb vs. TES groups; • *p* < 0.05, •• *p* < 0.01 comparing TES + Salb vs. TES + Salb + 4-AP groups; †† *p* < 0.01 comparing TES + Salb + 4-AP vs. TES; ¶ *p* < 0.05, ¶¶ *p* < 0.01 comparing TES + Salb + 4-AP + IBTX vs. TES groups.

As demonstrated in Figure 3H, TES (40 nM, for 48 h) increased the IK^+^ induced by depolarizing pulses and salbutamol (1000 nM) in ASM cells. However, this effect was blocked by indomethacin (1 µM) from −30 mV ahead. The area under the curve (AUC) analysis showed that indomethacin reduced the salbutamol-induced IK^+^ by 90.11% in myocytes preincubated with TES (Figure 8A), indicating that the nonselective COX inhibitor may directly regulate K^+^ channels activity. To identify the specific K^+^ channels subtypes involved in the TES-mediated enhancement of the salbutamol (1000 nM) response, cells were perfused with 4-aminopyridine (4-AP, 3 mM), a blocker of K_V_ channels. The resulting IK^+^ was nearly reduced by 4-AP from −50 mV ahead (Figure 8B). The subsequent addition of the selective blocker of BK_Ca_ channels, named iberiotoxin (IBTX, 100 nM), eliminated the residual salbutamol-induced IK^+^. The calculated AUC indicated that K_V_ and BK_Ca_ channels contributed 83.6% and 16.4%, respectively, to the TES-induced potentiation of the salbutamol response. Taken together, these data point to a predominant role of K_V_ channels, and to a lesser extent BK_Ca_ channels, as primary targets of indomethacin-mediated inhibition of salbutamol-evoked IK^+^.

## 3. Discussion

Our results reinforce our previous findings, indicating that prolonged exposure of ASM cells to physiological concentrations of TES enhances the IK^+^ elicited by salbutamol and theophylline [26,27]. In the present study, this TES-mediated potentiation was abolished by indomethacin, while ibuprofen and aspirin produced only partial inhibition. Thus, indomethacin exerts a dual action, inhibiting K^+^ channels activity and PG synthesis. In addition, the TES-induced enhancement of relaxation responses to both bronchodilators was also eliminated by indomethacin. Furthermore, indomethacin’s impact on salbutamol-induced IK^+^ correlates with the inhibitory effect of 4-AP and IBTX. Therefore, indomethacin likely reduces the activity of K_V_ and BK_Ca_ channels.

Salbutamol and theophylline are bronchodilators used in asthma patients to induce airway smooth muscle relaxation. Adrenergic agonists, such as salbutamol, stimulate the production of cAMP [32,33]. Theophylline is a methylxanthine that decreases the degradation of cAMP by inhibiting phosphodiesterases (PDEs) [33,34,35]. Additionally, these medications promote phosphorylation processes that lower intracellular Ca^2+^ levels [36,37]. In the present study, we found that salbutamol and theophylline induced the relaxation of the guinea pig ASM precontracted with histamine in a concentration-dependent manner (Figure 1A and Figure 2A). Moreover, prolonged exposure (48 h) to TES (40 nM) significantly enhanced the relaxation response for both drugs (Figure 1B and Figure 2B). This androgenic effect, implicating a genomic pathway, has been well documented in our previous work. In this context, the reason for the increased relaxation is attributed to TES (40 nM) upregulating K_V_1.2 and K_V_1.5 channels and β_2_ adrenergic receptor (β_2_-AR) in guinea pig ASM [26,27].

We have also investigated the direct effect of TES on guinea pig ASM tone. In this context, our previous studies demonstrated that adding cumulative concentrations of TES induced the relaxation of tracheal smooth muscle precontracted with carbachol or KCl [24,38]. Our research determined that the relaxation induced by TES was linked to the inhibition of L-type Ca^2+^ channels and store-operated Ca^2+^ channels. Additionally, evidence suggested a role for PGE_2_ in mediating TES-induced relaxation, as indomethacin partially reduced this effect in guinea pig ASM [24].

Considering these precedents, we sought to explore whether the potentiation of ASM relaxation induced by TES could involve the synthesis of prostaglandins, in addition to the already established upregulation of the β_2_-AR and K_V_ channels [26,27]. In support of this hypothesis, our present findings indicate that indomethacin impeded the action of the androgen on the augmented relaxation response induced by salbutamol and theophylline in tracheal tissues (Figure 1 and Figure 2). These observations suggest that TES may modulate the prostaglandin synthesis pathway through a genomic mechanism, given the extended incubation period (48 h). Indeed, previous studies have shown that TES influences COX expression and prostaglandin production across different tissues. For instance, in hamster Leydig cells [29] and in the porcine vas deferens epithelial cells [39], TES increases the expression of prostaglandin-endoperoxide synthase 2 (PTGS2, also known as COX-2), leading to increased production of PGF_2α_ [29]. Similarly, in rat kidney medullary tissues, TES increases the synthesis of PGE_2_ [40]. However, our recent investigations have demonstrated that TES did not augment the expression of either COX-1 or COX-2 in guinea pig tracheal tissues [28], suggesting that the modulation of PGs levels may occur via enzyme activity-dependent mechanisms rather than gene expression.

K^+^ channels are essential proteins that significantly contribute to the mechanistic action of methylxanthines and β_2_ adrenergic agonists [26,27,37,41]. The synthesis of cAMP, induced by these drugs, stimulates PKA, which is responsible for the phosphorylation of K^+^ channels, thereby increasing their opening. In airway preparations, several cell types, including ASM cells, epithelial cells, and fibroblasts, can synthesize prostaglandin PGE_2_ in response to physiological or pharmacological stimuli [42,43]. Given these multiple potential sources of PGE_2_ in organ bath preparations, we performed our electrophysiological studies using isolated tracheal smooth muscle cells, allowing us to evaluate the direct cellular effects of indomethacin and other NSAIDs on K^+^ channels without interference from epithelial- or fibroblast-derived prostanoids. Our results indicated that myocytes exposed to 40 nM TES exhibited increased IK^+^ in response to salbutamol (Figure 3E–H) and theophylline (Figure 4E–H) compared to the cells not exposed to the androgen. The enhancement of the IK^+^ was abolished by indomethacin (1 µM). Similarly, 1 µM ibuprofen and 10 µM aspirin partially reduced the action of TES on the IK^+^ induced by salbutamol (Figure 5 and Figure 6). We selected 1 µM indomethacin based on evidence showing that this concentration inhibits approximately 80% of PGE_2_ synthesis in human ASM cells, whereas 10 µM aspirin reduces PGE_2_ production by nearly 60% [44]. In contrast, data on the effects of ibuprofen on prostaglandin synthesis in ASM are limited. Existing studies employing ibuprofen in ASM models report concentrations ranging from 10 nM to 10 µM [45,46].

Up to this point in our study, we had not demonstrated the involvement of prostaglandins in the TES-induced potentiation of K^+^ currents, nor had we clarified the precise mechanism by which indomethacin inhibits this potentiation in response to salbutamol. However, previous research has shown that sustained elevations in intracellular cAMP levels, such as those triggered by prolonged exposure to β_2_-agonists like salbutamol, can stimulate the synthesis of PGE_2_ in airway smooth muscle cells [31]. Considering that TES enhances the expression of the β_2_-adrenergic receptor, it is plausible that this upregulation amplifies the cAMP signaling cascade in response to salbutamol, thereby promoting both the potentiation of K^+^ currents and increased PGE_2_ production.

To explore whether the ~40 min cumulative exposure to salbutamol during our concentration–response protocols could have activated prostaglandin synthesis, and thus contributed to the differential effects of NSAIDs, we conducted additional experiments using a single-concentration application of 1000 nM salbutamol (during 5 min, a period that does not allow for PG synthesis). Under these conditions, only indomethacin (1 µM) effectively blocked the TES-induced potentiation of salbutamol-evoked K^+^ currents, starting at −30 mV (Figure 7A), suggesting that this drug directly blocks these channels. In contrast, ibuprofen had no significant effect (Figure 7B), and aspirin incompletely reduced the TES-induced enhancement of K^+^ currents at 40 and 50 mV (Figure 7C), although these voltages are not physiologically relevant. However, this observation may still carry pharmacological implications; the effect is minimal compared to that of indomethacin.

In this sense, it has been documented that several COX inhibitors, including diclofenac sodium, celecoxib, and acetylsalicylic acid, can modulate K^+^ channels [47,48,49]. For instance, evidence indicates that diclofenac sodium enhances the conductance of BK_Ca_ channels and promotes the relaxation of acetylcholine-pre-contracted mouse ASM [47]. Furthermore, the selective inhibitor of COX-2, celecoxib (3 µM), has been shown to inactivate K_V_2.1 channels in rat cardiomyocytes, subsequently leading to reduced heart rate. This effect was also observed in HEK-293 cells [48]. In cochlear pericytes, acetylsalicylic acid exhibited an inhibitory effect on the outward K^+^ currents by blocking BK_Ca_ and K_V_ channel activity [49]. The structural properties of indomethacin and ibuprofen could partially explain their divergent effects on K^+^ channels. While ibuprofen is a propionic acid derivative, indomethacin is derived from an acetic acid [50]. Notably, the interaction of acetic acid with K^+^ channels has been previously demonstrated in guinea pig detrusor smooth muscle [51]. Moreover, we found that indomethacin reduced 90.11% of the increased IK^+^ induced by salbutamol in myocytes chronically exposed to TES (Figure 8A). In the ASM, the K_V_ and BK_Ca_ channels represent the primary proteins involved in the IK^+^ evoked by salbutamol and theophylline [26,27,52]. To identify the K^+^ channel subtypes that may be regulated by indomethacin, we utilized 4-AP and IBTX. As illustrated in Figure 8B, in myocytes exposed to TES, K_V_ channels contribute approximately 83.6%, while BK_Ca_ channels represent 16.4%. These results indicate that indomethacin primarily eliminates the androgen effect by inhibiting K_V_ channels and, to a lesser extent, the BK_Ca_ channels. These findings contribute to the existing research concerning the influence of NSAIDs on K^+^ channels and indicate that indomethacin, but not ibuprofen or aspirin, blocks mainly K_V_ currents in ASM cells.

It has also been shown that long-term NSAID treatment can reduce the expression of certain K_V_ channel subtypes, such as K_V_1.4 and K_V_1.6 [53]. However, those effects were reported under conditions involving 72 h drug exposure. In contrast, our experiments used much shorter treatment durations, from 10 to 40 min in isolated ASM cells during patch-clamp recordings, and 2 h in tracheal ring assays. Such brief exposure times are unlikely to induce transcriptional downregulation of K_V_ channels. This is supported by studies showing that downregulation of K^+^ channel expression typically requires long periods (≥48 h) [54]. Although we did not directly assess protein levels of K_V_ channels, the rapid and significant inhibition of K^+^ currents observed within 10–15 min of indomethacin exposure (Figure 3, Figure 4 and Figure 7) strongly suggests an acute pharmacological effect on channel function rather than a genomic or proteomic alteration.

In the context of asthmatic disease, some patients have demonstrated a non-immunoglobulin E (IgE) hypersensitivity reaction to NSAIDs [55]. In particular, the intake of aspirin has been observed to provoke bronchospasm [56,57,58,59]. The mechanism through which aspirin exacerbates asthma has been associated with an increased production of cysteinyl leukotrienes (LTs) and a decreased synthesis of PGE_2_ [60,61]. Additionally, an increase in the number of Group 2 innate lymphoid cells (ILC2s) has been reported in patients with aspirin-exacerbated respiratory disease (AERD) following aspirin challenges [62]. The function of ILC2s may be essential in determining the clinical characteristics of NSAID-induced reactions in AERD, as type 2 inflammation represents a significant component of the condition [63]. Regarding indomethacin, a daily dose of 200 mg of this medication did not result in the aggravation of asthma after exercise or antigen challenge. However, the authors of that study did not perform a sex-based analysis [64]. Conversely, the use of ibuprofen may lead to asthma exacerbations in pediatric patients previously diagnosed with asthma, although the underlying mechanism has not been elucidated [65]. In light of these observations, it could be inferred that in the context of AERD, the blockade of K^+^ channels is not involved. However, our hypothesis proposes that indomethacin and ibuprofen, to a lesser extent, can block these channels in the ASM.

Collectively, our findings demonstrate that indomethacin exerts a dual effect: it not only inhibits prostaglandin synthesis but also directly blocks K^+^ channels. This combined action likely explains the more pronounced inhibitory effect of indomethacin compared to other NSAIDs. This leads to a significant reduction in the TES-mediated enhancement of relaxation induced by salbutamol and theophylline in airway smooth muscle. Interestingly, it has been observed that through genomic mechanisms, TES evokes an anti-inflammatory state and promotes a more relaxed condition of smooth muscle [26,27,66,67], thereby serving as a protective factor against asthma. Therefore, it is advisable to exercise caution when prescribing indomethacin to young males with asthma, as the protective role of TES may diminish, potentially resulting in an exacerbation of asthma symptoms. Furthermore, our data suggest that even though ibuprofen and aspirin do not acutely block K^+^ currents to the same extent as indomethacin, their ability to inhibit prostaglandin synthesis could still interfere with the therapeutic actions of salbutamol.

## 4. Materials and Methods

### 4.1. Animals

Male Hartley guinea pigs, aged four to six weeks and weighing between 350 and 400 g, were used for this study. The animals were bred in our institutional animal facilities under conventional conditions, which included free access to food and sterile water, filtered conditioned air, a temperature of 21 ± 1 °C, and 50–70% humidity. The Scientific and Bioethics Committees approved the experimental procedure at the Facultad de Medicina of the Universidad Nacional Autónoma de México (UNAM FM/DI/003/2020). For conducting the experiments, we adhered to the Guide for the Care and Use of Vertebrate Animals, as established by the American Physiological Society and the National Institutes of Health Guidelines for the Care and Use of Laboratory Animals.

### 4.2. Organ Baths

Guinea pigs were euthanized using intraperitoneally administered sodium pentobarbital (50 mg/Kg) and then exsanguinated. The tracheas were removed from each guinea pig and dissected on a silica plate containing Krebs solution for approximately 30 min. Krebs solution consisted of the following (mM): 118 NaCl, 25 NaHCO_3_, 4.6 KCl, 1.2 KH_2_PO_4_, 1.2 MgSO_4_, 2 CaCl_2_, and 11 glucose. The solution was continuously bubbled with a carbogen mixture of 95% O_2_ and 5% CO_2_, maintaining a pH of 7.3 to 7.4. The tracheas were stripped of epithelium and connective tissue and washed three times to remove any remaining cellular debris and blood. They were then cut into eight rings. Each ring was kept individually in Eppendorf tubes containing bubbled Krebs solution (previously bubbled with 95% O_2_ and 5% CO_2_) and maintained at 9 °C for 48 h. Some tissues were incubated with 40 nM TES [26].

After the 48 h incubation, each ring was hung on wire hooks placed in tissue baths (EmkaBath system, Emka Technologies, Paris, France) filled with 10 mL of Krebs solution at 37 °C, which was aerated with 5% CO_2_ in oxygen to maintain a pH of 7.3–7.4. It is important to note that none of the organ bath chambers were added with TES at this point. The hooks were attached to an isometric force transducer (Emka Technologies), and the rings were set to a resting tension of 1 g for 1 h before the experimental procedures began. Changes in isometric force were recorded using the software Datanalyst 2.7.0.25 (Emka Technologies). After the equilibrium period, the tissues were stimulated with KCl (60 mM) three times to optimize the contractile apparatus. Some tissues were treated with indomethacin (1 µM) to inhibit COX-1 and COX-2 activity for 1 h. Afterward, all tissues were pre-contracted with 10 µM histamine (His). Once a plateau was reached, cumulative curves of salbutamol or theophylline were constructed to evaluate the relaxation of ASM. Indomethacin was maintained during histamine and salbutamol stimulation.

### 4.3. Patch-Clamp Studies

Cultured guinea pig ASM cells were used for patch clamp recordings. The tracheas were dissected to remove fat, connective tissue, and epithelium, and the ASM was collected. This tissue was placed in 5 mL of Hanks’ solution (GIBCO, Waltham, MA, USA) containing 2 mg of L-cysteine and 0.05 U/mL of papain (Worthington Biochemical, Lakewood, NJ, USA) for 10 min at 37 °C. Afterward, the enzyme was washed out using Leibovitz solution (L-15, GIBCO), and the tissue was incubated in Hanks’ solution containing 1 mg/mL of collagenase type I and 1 mg/mL of collagenase type II (Worthington Biochemical) for an additional 10 min. Next, the tissue was mechanically disaggregated using a Pasteur pipette by aspirating and releasing 30 times, and then the tissue was incubated again at 37 °C. After another 10 min, the tissue was mechanically separated again, and the enzymatic activity was halted with L-15. The cells were centrifuged for 5 min at 600 rpm, and this procedure was repeated one more time. Finally, the supernatant was discarded, and the cell pellet was resuspended in minimal essential medium (MEM, GIBCO) containing 2 mM L-glutamine, 10 mM glucose, 10 μg/mL streptomycin, 10 U/mL penicillin, and 5% fetal bovine serum. The cells were then placed on coverslips pre-coated with collagen from rat tails and cultured for 48 h in a 5% CO_2_ incubator (Panasonic, Newark, NJ, USA).

ASM cells on the coverslips were submerged and settled into a 2 mL chamber mounted on an inverted microscope (Zeiss model IP03, Jena, Germany). The chamber was continuously perfused with a specific external solution for measuring K^+^ currents at a 1.5–2 mL/min rate. The composition of the external solution was as follows (mM): 0.1 niflumic acid, 0.5 MgCl_2_, 1 CaCl_2_, 1.2 KH_2_PO_4_, 3 NaHCO_3_, 5 KCl, 10 HEPES, 10 glucose, and 130 NaCl. The pH of the external solution was adjusted to 7.4 using NaOH. Patch pipettes were made from borosilicate capillaries (1B200F-6 glass, Word Precision Instruments, Sarasota, FL, USA) using a micropipette puller (P-87, Sutter Instruments Co., Novato, CA, USA). These pipettes were filled with an internal solution containing (mM): 0.1 leupeptin, 0.1 GTP, 1 EGTA, 5 NaCl, 5 HEPES, 5 ATP, and 140 K^+^ gluconate, with the pH adjusted to 7.3 using KOH. The pipette resistance before cell attachment ranged from 2 to 5 MΩ. An amplifier (Axopatch 200A, Axon Instruments, San Jose, CA, USA) was used to measure and record outward K^+^ currents. Whole-cell currents were filtered at 1–5 kHz, digitized (Digidata1440A, Axon Instruments) at 10 kHz, and stored on a computer for subsequent analysis using software pClamp, version 10.2. Patch-clamp studies were performed at room temperature (approximately 22 °C).

To assess K^+^ currents, a series of depolarizing pulses ranging from −60 to +50 mV in 10 mV increments with a holding potential of −60 mV were applied to clamped cells for 500 ms at a frequency of 1 Hz. Following the control depolarizing pulse protocol, myocytes were perfused with increased concentrations of salbutamol or theophylline and subjected to depolarizing pulses. Some myocytes were treated with indomethacin (1 µM), ibuprofen (1 µM), or acetylsalicylic acid (10 µM) 10 min before adding salbutamol or theophylline. To identify the K^+^ channels implicated, myocytes were treated with 3 mM of 4-aminopyridine (4-AP, which blocks delayed rectifier K^+^ channels) or 100 nM of iberiotoxin (IBTX, a specific blocker of high-conductance Ca^2+^-activated K^+^ channels). The variations in K^+^ currents were assessed by measuring the peak current for each voltage applied.

### 4.4. Drugs and Chemicals

Testosterone (17β-hydroxy-4-androsten-3-one, TES), histamine, salbutamol, theophylline (1,3-dimethylxanthine), indomethacin, acetylsalicylic acid, and ibuprofen were all purchased from Sigma Chem. Co. (St. Louis, MO, USA). 4-Aminopyridine was obtained from Research Chemical LTD (Word Hill, MA, USA), and iberiotoxin was sourced from Enzo Life Sciences (Farmingdale, NY, USA). TES was diluted in absolute ethanol, with the highest concentration being 0.1% *v*/*v* of the vehicle.

### 4.5. Statistical Analysis

In organ bath studies, the relaxation of tracheal smooth muscle caused by salbutamol or theophylline was evaluated using the inhibitory concentration of 50% (IC_50_) and the maximum relaxation. Each cumulative concentration–response curve was used to calculate the IC_50_, which was computed by straight-line regression as −Log [M] using ED50 plus v1.0 software. The analysis involved repeated-measures analysis of variance, along with Dunnett’s multiple comparison test. K^+^ currents at each voltage step were analyzed using one-way analysis of variance with Dunnett’s tests or repeated-measures followed by Student–Newman–Keuls’ tests. The area under the curve (AUC) of the K^+^ currents was determined using SigmaPlot software v12.0. In all experimental procedures, each value of ”*n*” is representative of a distinct animal. The manuscript and accompanying figures present data as mean ± standard error of the mean (SEM). Statistical significance was established at a threshold of *p* < 0.05.

## 5. Conclusions

In summary, our study demonstrates that indomethacin directly blocks the activity of K_V_ and BK_Ca_ channels and PGE_2_ production in guinea pig ASM cells chronically exposed to TES. This mechanism abolishes the androgen-enhanced ASM relaxation induced by salbutamol and theophylline. Caution should be taken when prescribing these medications to asthmatic young males.

## Data Availability

Data are available upon request.

## Abbreviations

The following abbreviations are used in this manuscript:

ASA	Acetylsalicylic acid
ASM	Airway smooth muscle
COX	Cyclooxygenase
Ibu	Ibuprofen
IK^+^	Outward K^+^ current
